# Bedside Ultrasound Identification of Infectious Flexor Tenosynovitis in the Emergency Department

**DOI:** 10.5811/westjem.2015.1.24474

**Published:** 2015-03-06

**Authors:** Kevin Padrez, Jennifer Bress, Brian Johnson, Arun Nagdev

**Affiliations:** * University of California, San Francisco, School of Medicine, San Francisco, California; † Tufts University, School of Medicine, Boston, Massachusetts; ‡ Alameda Health System, Emergency Department, Highland Hospital, Oakland, California

## Abstract

Infectious flexor tenosynovitis (FTS) is a serious infection of the hand and wrist that can lead to necrosis and amputation without prompt diagnosis and surgical debridement. Despite the growing use of point-of-care ultrasound (POCUS) by emergency physicians there is only one reported case of the use of POCUS for the diagnosis of infectious FTS in the emergency department setting. We present a case of a 58 year-old man where POCUS identified tissue necrosis and fluid along the flexor tendon sheath of the hand. Subsequent surgical pathology confirmed the diagnosis of infectious FTS.

## CASE REPORT

A 58-year-old man with a history of diabetes, hypertension and end-stage renal disease on hemodialysis presented to the emergency department (ED) for one day of right hand pain and swelling. The pain was diffuse (hand, wrist and distal forearm), and occurred with any movement. The patient denied any trauma, numbness, weakness, fever, or chills. The patient’s vital signs were temperature of 99.0 degrees Celsius, heart rate of 91 beats per minute, blood pressure of 131/76mmHg, and respiratory rate of 18 breaths per minute. On examination, the right hand was more swollen than the left. He held the hand in flexion, with increased pain on both flexion and extension of the fingers and wrist. There was no crepitus, warmth, or erythema, and distal pulses were intact. The patient’s neurologic examination was normal. His right upper extremity had a matured vascular shunt, which had a strong bruit and thrill.

**Figure 1:**
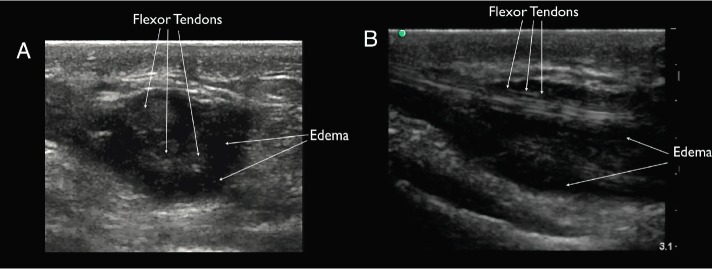
Ultrasound findings of flexor tenosynovitis. Ultrasound image in transverse view (A) and longitudinal view (B) showing increased anechoic edema and debris in the flexor tendon sheath of the right wrist consistent with flexor tenosynovitis. In the correct clinical scenario, these findings are consistent with the infectious form of flexor tenosynovitis.

Laboratory results revealed a lactate of 1.9mmol/L and a normal white blood cell count. On radiograph of the right hand and wrist, there were degenerative changes and mild soft tissue swelling, but no acute fractures, subluxations, or focal erosion. ED providers performed point-of-care ultrasound (POCUS) using a high-frequency (13MHz to 6MHz) linear transducer (Sonosite M-Turbo^TM^, Bothell WA) that demonstrated a moderate amount of fluid and hypoechoic material in the flexor tendon sheath (as compared to the contralateral hand) (Figure 1, Video 1 and Video 2).

Orthopedics was consulted. The patient was started on broad-spectrum antibiotics and taken to the operating room. Intraoperatively, purulent fluid was found extensively along the flexor tendon sheath of the fifth phalange extending to the common flexor sheath of the wrist. Surgical fluid cultures were positive for *Staphylococcus aureus* with the patient requiring a two-week course of inpatient antibiotic therapy.

## DISCUSSION

Infectious tenosynovitis occurs when purulent fluid collects between the visceral and parietal layers of a tendon, caused by infection via direct inoculation (trauma), hematologic spread, or contiguous spread from adjacent tissues.^1^,^2^ The most common location for infectious flexor tenosynovitis is in the hand and wrist and colloquially described as FTS. In the hand, the flexor digitorum superficialis and flexor digitorum profundus tendons lie within the common flexor sheath, which courses through the carpal tunnel into the palm of the hand.

FTS is often diagnosed clinically, using Kanavel’s four cardinal signs initially described in 1912.^3^ Kanavel’s signs are intense pain along the course of a tendon with attempted extension of a partly flexed digit, the finger held in flexion for comfort, swelling over the entire finger, and percussion tenderness over the course of the tendon sheath.^4^ Clinical examination is thought to have high specificity and positive predictive value for infectious FTS. However, a negative exam does not rule out infectious FTS.^5^,^6^ Infectious FTS is treated with empiric antibiotic therapy, as well as emergent surgical debridement and drainage. Early diagnosis and prompt surgical debridement is essential. Delay to diagnosis leads to the local spread of infection to adjacent tissue and bursa, compartment syndrome, and necrosis. Risk factors for poor prognosis include age over 43, diabetes, peripheral vascular disease, renal failure, digital ischemia or subcutaneous purulence.^2^ Patients with septic necrosis of the tendon sheath may require amputation of the affected digit.

**Figure 2:**
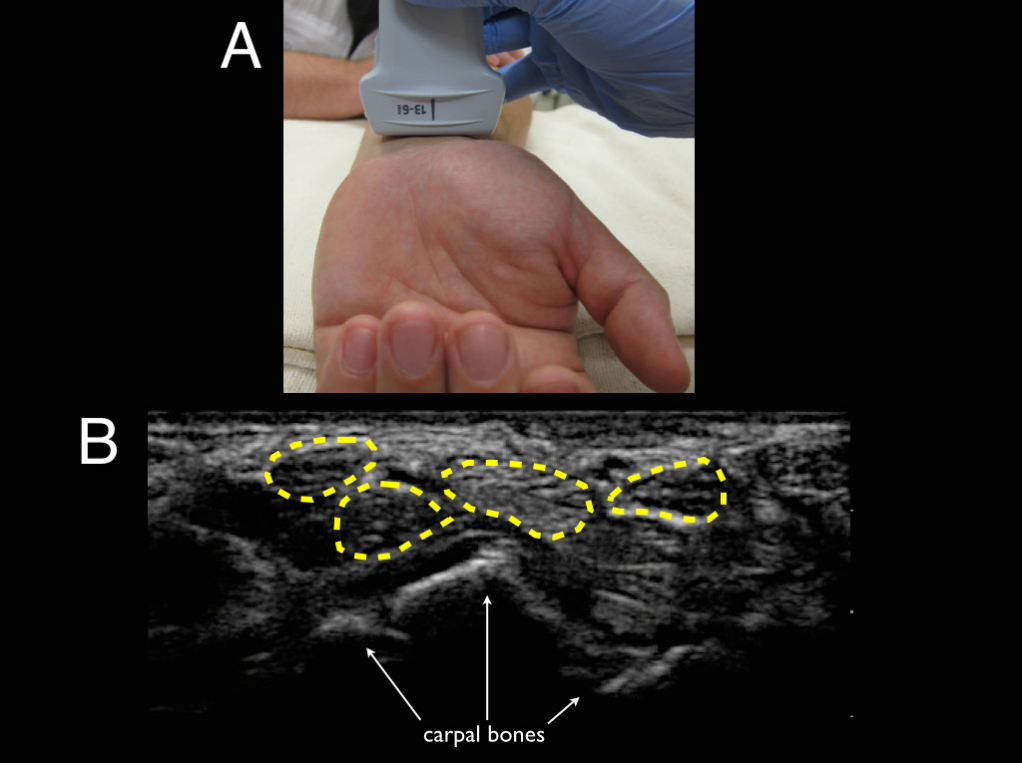
Ultrasound technique and visualization. (A) Appropriate placement of the linear transducer (13MHz to 6MHz) at the wrist crease in transverse plane to evaluate for flexor tenosynovitis. (B) Ultrasound image in transverse view showing normal flexor tendons (highlighted in yellow) with no surrounding edema. The flexor tendons should lie anterior to the carpal bones identified by arrows for reference.

Hand radiographs are often obtained in cases of suspected infectious FTS to look for trauma, retained foreign bodies, or bone erosion. However, radiograph cannot make the diagnosis of infectious FTS. Magnetic resonance imaging (MRI) and ultrasound are better imaging modalities for the diagnosis.^6^,^7^ While MRI is not typically available in a prompt fashion in the ED, ultrasound is often available to ED providers.^8^ Ultrasound has been shown to be more sensitive than clinical exam for detecting tenosynovitis.^5^,^9^ However, there is only one case in the literature describing the use of ED POCUS to diagnosis infectious FTS.^10^ Common ultrasound findings for FTS are hypoechoic or anechoic edema with a potentially thickened tendon sheath, as well as the presence of abnormal hypoechoic material within the synovial sheath.^9^,^11^ Typically this can be performed using a linear probe and rocking the transducer to achieve perpendicular orientation of the tendon sheath (not the skin) in the transverse and longitudinal plane (Figure 2).^9^

In the ED setting, we recommend using POCUS in conjunction with the clinical examination to evaluate for suspected infectious FTS. The ability of POCUS to diagnose infectious FTS from inflammatory FTS is unknown. The clinical context is critical when incorporating POCUS to increase diagnostic certainty and perhaps expedite definitive care. While no standard definition exists, the sonographic finding of increased hypoechoic/anechoic fluid or material in the flexor tendon sheath is highly suspicious for infectious FTS in the appropriate clinical scenario. However, a negative POCUS study for FTS should not discourage aggressive consideration of the diagnosis.

In conclusion, we report a case that introduces the use of POCUS to identify infectious FTS in the ED setting. Oftentimes, history and clinical examination cannot rule out the diagnosis. POCUS may be an ideal adjunct for the ED physician in the evaluation of a patient with suspected infectious FTS.

## Figures and Tables

**Video 1:** Ultrasound video in transverse view showing increased anechoic edema and debris in the flexor tendon sheath of the right wrist consistent with infectious flexor tenosynovitis.

**Video 2:** Longitudinal view showing increased anechoic edema and debris in the flexor tendon sheath of the right wrist consistent with infectious flexor tenosynovitis.
